# A Curious Case of Purple Chromaturia

**DOI:** 10.7759/cureus.77235

**Published:** 2025-01-10

**Authors:** Richa Dhakal, George S Zacharia, Nismat Javed, Puja K. C., Misbahuddin Khaja

**Affiliations:** 1 Internal Medicine, BronxCare Health System, Bronx, USA; 2 Pulmonary Critical Care, BronxCare Health System, Bronx, USA

**Keywords:** chromaturia, cyanide toxicity, hydroxocobalamin, management, purple urine

## Abstract

Hydroxocobalamin, an antidote for cyanide toxicity, is known for its safety and efficacy. A notable side effect is dark red to purple chromaturia, sometimes mimicking hematuria. We report the case of a middle-aged female exposed to smoke inhalation following a house fire, who was treated on-site by emergency medical services with oxygenation and intravenous hydroxocobalamin and developed purple urine shortly after admission. Hematuria, hemolysis, rhabdomyolysis, and renal impairment were excluded with appropriate workup. The discoloration resolved spontaneously over five weeks without intervention, consistent with hydroxocobalamin-induced chromaturia. While benign and self-limiting, this phenomenon may cause diagnostic confusion and patient concern. Awareness of this side effect can prevent unnecessary investigations and reassure patients about its transient nature.

## Introduction

Hydroxocobalamin is used as an antidote in suspected cases of cyanide toxicity. Typically, a benign molecule, administered intravenously, binds with cyanide to form non-toxic cyanocobalamin, which is subsequently excreted through urine; however, it is known to cause a staring dark red to purple chromaturia, which may last for several weeks [1,2]. Chromaturia is usually a sign of an underlying disease and might not necessarily be associated with symptoms [2,3]. The sign can be suggestive of multiple underlying pathologies, for example, alkaptonuria, which can lead to black discoloration of urine upon standing [4]. Chromaturia, however, has little, if any, toxic effects but evokes concern in patients and clinicians, as it closely mimics hematuria. The sign serves the purpose of focusing on specific differential diagnoses that must be isolated for example familial disorders or porphyrin [3,4]. Once alternate causes of urinary discoloration are excluded, such chromaturia requires no specific treatment and clears off by itself over a few weeks.

## Case presentation

A 59-year-old female with a history of bronchial asthma was brought by emergency medical services (EMS) with shortness of breath following smoke inhalation from a house fire. On site, she was found unconscious and received emergency management by the EMS personnel. Upon arrival at the emergency department, she had tachycardia (pulse rate of 123 beats/minute) and tachypnea (respiratory rate of 36 breaths/minute). Her physical examination revealed diffuse wheezing. In view of her breathing effort, she was started on bilevel positive airway pressure (BiPAP). Initial lab investigations revealed mild hypokalemia and metabolic acidosis (Table 1).

**Table 1:** Lab investigations

Investigation	Value	Reference
White blood cells (k/uL)	9.8	4.8-10.8
Hemoglobin (g/dL)	13.4	12.0-16.0
Platelets (k/uL)	259	150-400
Sodium (mEq/L)	138	135-145
Potassium (mEq/L)	3.4	3.5-5.0
Bicarbonate (mEq/L)	21	24-30
Blood urea nitrogen (mEq/L)	15	6-20
Creatinine (mEq/L)	1.2	0.5-1.5

**Figure 1:**
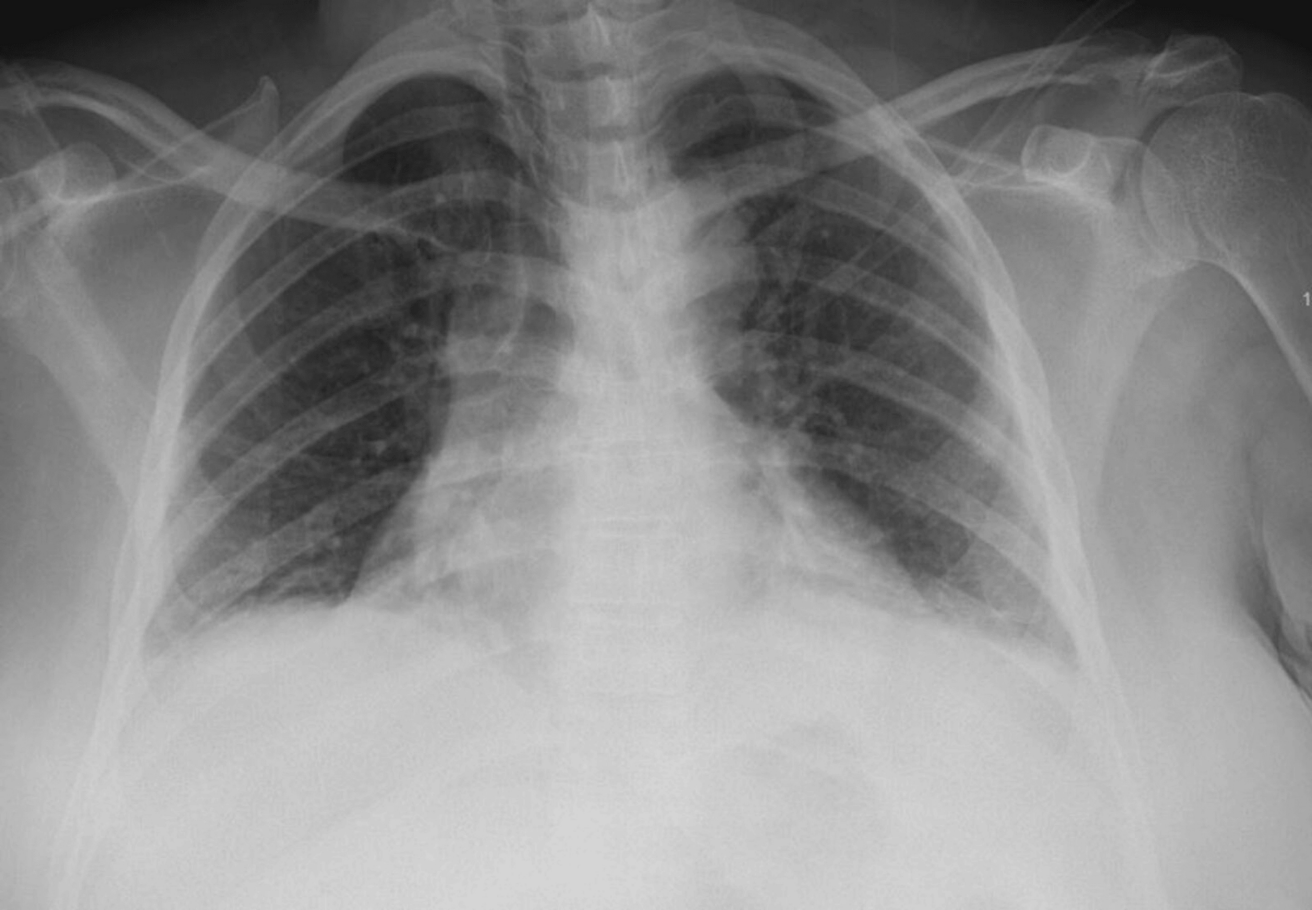
Portable chest X-ray showing atelectatic changes

She had lactic acidosis (5.1 mmol/L) but a normal carboxyhemoglobin level. Chest X-ray suggested bronchitis and mild bilateral lower lobe atelectasis (Figure 1).

**Figure 2:**
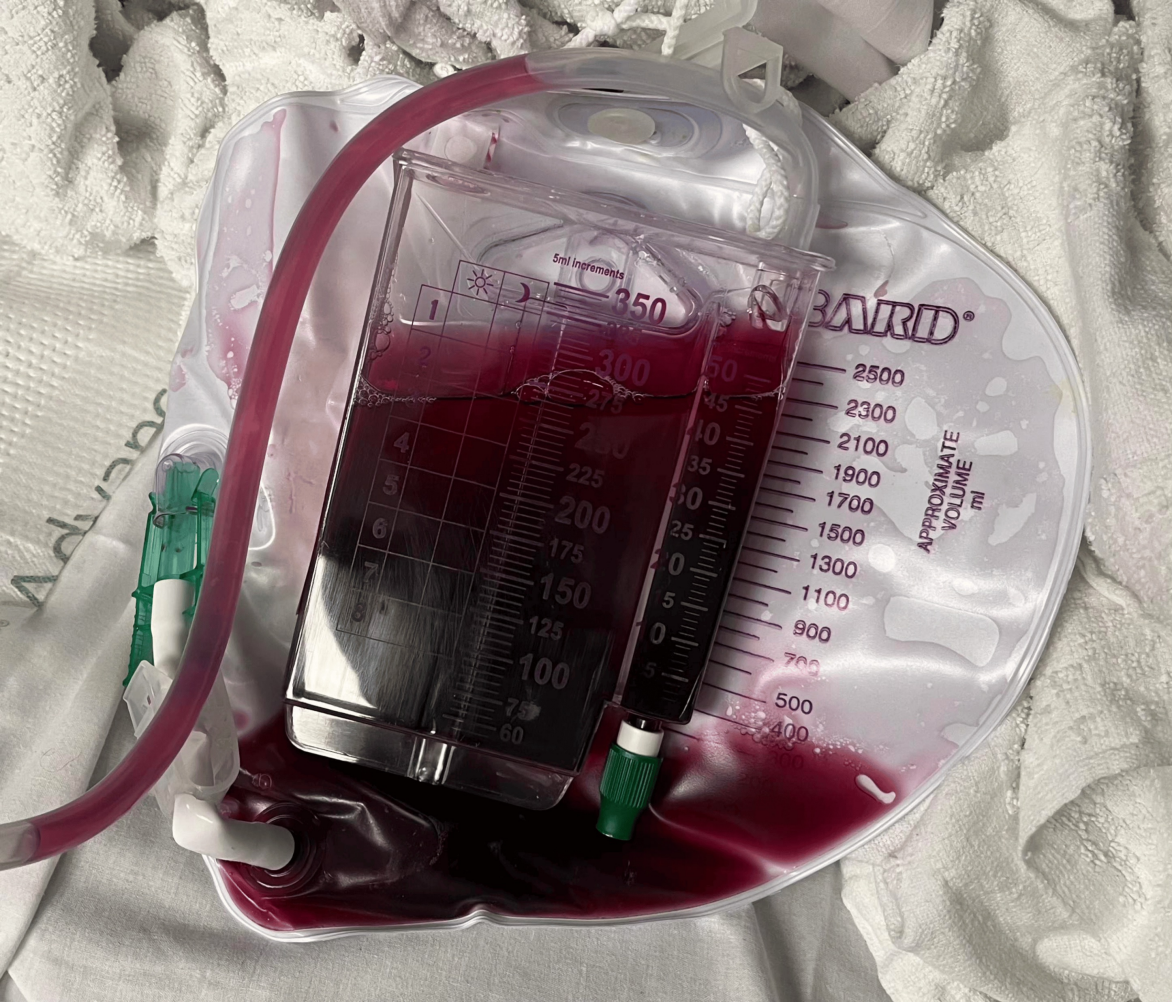
Indwelling catheter showing purple to purple-red urine

She was managed with the continuation of BiPAP, bronchodilators, systemic steroids, and supportive and symptomatic measures, which improved her respiratory distress. She had already been administered oxygen and intravenous medications on-site by paramedics. However, the following day, she noticed urinary discoloration, red to purplish urine, but she had no other urinary symptoms (Figure 2).

Urinalysis did not reveal hematuria, hemoglobinuria, or pyuria. An indwelling urinary catheter was placed, which drained purplish-red urine. She had no hepatic or renal dysfunction at baseline or during the entire course of hospitalization, nor did she had any evidence of rhabdomyolysis. She declined the use of any medications or recreational drugs except for inhalational bronchodilators and steroids. She was managed additionally for asthma exacerbation secondary to smoke inhalation. The otolaryngology team also evaluated her for any signs of inhalation injury from smoke and she was cleared for discharge.

During her hospital course, she continued to be asymptomatic, and by the time of discharge, her urine started to clear. On a follow-up tele-consult, she reported complete clearance of the urine by around two weeks of the inciting event.

## Discussion

Chromaturia refers to urine discoloration beyond its physiological clear to pale yellow hue. The color of urine depends on its concentration and constituents. Urobilinogen in the urine, derived from bilirubin, gets oxidized to urobilin upon exposure to air and imparts the physiological yellow tint to urine. [3] A myriad of drugs, diets, and ailments have been linked to chromaturia. It can be benign, like pink to reddish with beets and berries, a sign of underlying pathology, deep yellow to brown discoloration such as in advanced liver disease, cola-colored such as in hematuria, or deep brown to black such as in alkaptonuria [4]. Urine color is, hence, considered a key component of urinalysis in the history of medicine. A concise list of the causes of chromaturia is summarized in Table 2 [5,6].

**Table 2:** Causes of chromaturia

Urine Color	Potential Causes	Underlying Pathologies
Red/pink	Hematuria, hemoglobinuria, myoglobinuria, porphyrins, diet, drugs	Urinary tract infections, kidney stones, tumors, intravascular hemolysis, rhabdomyolysis, rifampin, phenazopyridine
Brown/tea	Bilirubinuria, alkaptonuria, certain foods/medications	Liver disease or biliary obstruction, nitrofurantoin, metronidazole
Orange	Dehydration (concentrated urine), medications, excess beta-carotene	Phenazopyridine, rifampin, high dietary intake of carrots
Blue/green	Amitriptyline, indomethacin, propofol, methylene blue, triamterene, bacterial infections, rare inherited disorders	“Blue diaper syndrome” (familial hypercalcemia), Pseudomonas infection may cause green discoloration
Purple	Purple urine bag syndrome, porphyrins	Usually associated with *Klebsiella* or *Escherichia coli *producing indigo and indirubin
Black/dark	Alkaptonuria, melanuria	Melanogens that oxidize/darken upon standing

Apart from direct and indirect thermal injuries, house fire exposures can result in carbon monoxide and cyanide inhalational toxicities. A summary of the cases and their features is shown in Table 3.

**Table 3:** Cases of chromaturia

Author	Age/Gender	Symptoms	Labs	Management	Outcome
Wong et al. [7]	49/male	Asymptomatic	Carboxyhemoglobin-20.5%, urinalysis showing purple urine	Hydroxocobalamin	Alive and resolution in few days
Hardin et al. [8]	47/male	Hypotension, cyanosis, depressed mental status,	Chocolate colored arterial blood, urinalysis showing purple urine, methemoglobin 66%	Methylene blue, hydroxocobalamin	Alive and resolution in 1 week
Geraci et al. [9]	92/female	Shortness of breath requiring intubation	Urine showing deep red color converted to purple later	Mechanical ventilation, hyperbaric oxygen therapy, hydroxocobalamin	Death and resolution of chromaturia in 8 days
Cescon et al. [10]	54/female	Altered mental status, hypotension, and evidence of inhalational injury	Urine showing red color, carboxyhemoglobin level was 29% and lactate level was 16 mmol/L	Supplemental and hyperbaric oxygen, hydroxocobalamin	Not clear
Koratala et al. [11]	70/female	Underwent incisional hernia repair and adhesiolysis that was complicated by bowel injury, leading to peritonitis and refractory septic shock	Urinalysis showing red urine	Multiple vasopressors and hydroxocobalamin	Death

Most of the cases were between 40 and 95 years of age, which is comparable to our patient [7-11]. Most of the patients had presented in an unstable state and died as a result. However, the presence of colored urine did not reflect the outcome or the etiology, rather a transient impact of treatment [9-11]. Most of the cases died, as seen in Table 3.

Oxygen therapy at the highest possible concentration is the cornerstone of managing carbon monoxide poisoning [12]. In suspected cyanide toxicity, the pre-hospital management strategy includes oxygen inhalation together with specific antidotes; the most frequently utilized antidote is hydroxocobalamin. It works almost instantaneously upon intravenous administration by covalently binding with cyanide to form cyanocobalamin [1,7].

Owing to its safety profile, hydroxocobalamin is utilized frequently as a pre-hospital antidote in cases of suspected cyanide toxicity. However, it may impart a dark red-to-purple hue to plasma, urine, tears, sweat, and skin. This chromaturia sometimes mimics hematuria and raises concerns among patients and clinicians. Although hydroxocobalamin-associated discoloration is benign and self-resolving, it may last up to five weeks [2]. Also, it is reported to interfere with CO-oximetry, often resulting in falsely higher carboxyhemoglobin concentrations [13].

Our patient was administered oxygen and intravenous medications on-site by paramedics, retrospectively identified as hydroxocobalamin, before arrival at the hospital. She had deep red to purplish urine by the end of the first day of admission, presumably related to hydroxocobalamin exposure. She denied the use of other medications and had no features of hemolysis, rhabdomyolysis, or renal failure. Urinalysis revealed no heme or erythrocytes. The exposure to hydroxocobalamin, lack of alternate causes for chromaturia, benign nature, and spontaneous clearance over time suggest the diagnosis of hydroxocobalamin-induced chromaturia in this case.

## Conclusions

Hydroxocobalamin-induced chromaturia is a benign and self-limiting phenomenon that can mimic hematuria and result in patient anxiety. Awareness of this side effect is essential for clinicians potentially involved in the management of cyanide toxicity, as it prevents misdiagnosis and facilitates appropriate patient reassurance. The case highlights the importance of understanding the pharmacological profile of hydroxocobalamin, ensuring confidence in its use as a life-saving antidote. Essentially, a very important part of counseling comes into play when explaining to patients regarding their care; patients who receive hydroxocobalamin should be counseled regarding this change in urine color and its implication for future management.
